# Using Bioelements Isotope Ratios and Fatty Acid Composition to Deduce Beef Origin and Zebu Feeding Regime in Cameroon

**DOI:** 10.3390/molecules26082155

**Published:** 2021-04-08

**Authors:** Matteo Perini, Mohamadou Bawe Nfor, Federica Camin, Silvia Pianezze, Edi Piasentier

**Affiliations:** 1 Fondazione Edmund Mach, 38098 San Michele All’Adige, Trento, Italy; federica.camin@unitn.it (F.C.); silvia.pianezze@fmach.it (S.P.); 2 Department of Rangeland, Animal Nutrition and Livestock Infrastructures, Sub Department of Animal Nutrition, Ministry of Livestock, Fisheries and Animal Industries (MINEPIA), Yaoundè, Cameroon; nformohamadou42@yahoo.com; 3 Centre Agriculture Food Environment C3A, University of Trento, 38098 San Michele All’Adige, Trento, Italy; 4 Department of Agricultural, Food, Environmental and Animal Sciences DI4A, University of Udine, 33100 Udine, Italy; Edi.Piasentier@uniud.it

**Keywords:** Cameroon beef, stable isotope analysis, meat color, PUFA and SFA

## Abstract

The purpose of this study was to address the lack of knowledge regarding the stable isotopic composition of beef from zebu cattle reared in tropical Africa. Sixty beef carcasses belonging to the most common zebu breeds (Goudali, white Fulani, and red Mbororo) were selected and classified according to their subcutaneous fat color (white, cream or yellow). The stable isotope ratios of five bioelements—H, O, C, N, and S—in muscle fractions and the fatty acids composition were analyzed. Zebu meat from Cameroon shows peculiar δ^13^C values, related to the almost exclusive intake of grazed tropical grasses with photosynthetic cycle C4. It also shows δ^2^H and δ^18^O values higher than those reported in other areas of the world and correlated with the isotopic composition of animal drinking water. The white subcutaneous fat (“white type”) zebu showed higher δ^2^H and lower δ^13^C than the “yellow type”, that is correlated with a higher content of polyunsaturated fatty acid (PUFA) and a lower amount of saturated fatty acid (SFA) and monounsaturated fatty acid (MUFA). Multielement analysis seems to provide promising results for tracing the regional origin of Cameroon beef and some aspects of the livestock system, such as the nutritional status of the animals.

## 1. Introduction

Zebu (*Bos taurus indicus*) is a subspecies of domestic cattle, originally coming from South Asia. About 75 breeds of zebu are known and they are evenly divided between Africa and India [1]. Zebu are characterized by a fatty hump on their shoulders, a large dewlap, and sometimes by drooping ears. Compared to the taurine cattle (*Bos taurus taurus*), also known as the European cattle, zebu are well adapted to the hot and dry environment of the tropics and show an appreciable tolerance to endemic diseases [2,3]. Zebu are used as hauling and riding animals, whereas dairy and beef cattle are also source of byproducts such as hide and dung for fuel and manure [1]. 

**Figure 1:**
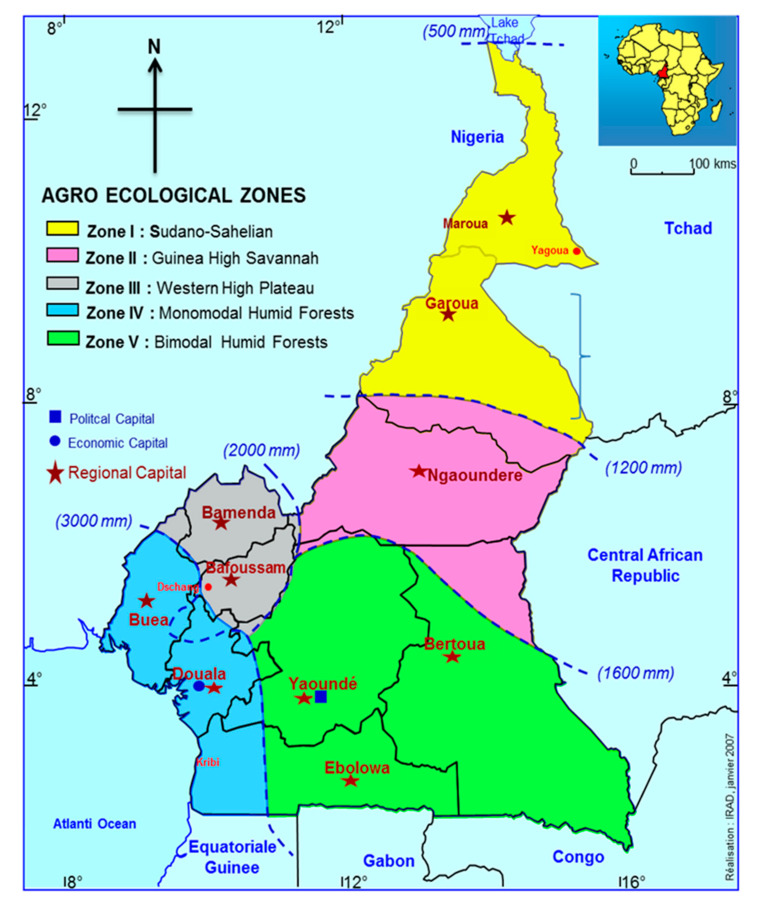
Map of Cameroon showing the five agroecological zones.

Having an estimated population of 6,500,000 head of cattle reared in five ecological zones (Figure 1) [4], Cameroon is one of the main regional providers of beef and other cattle-derived products [5,6] of the Central Africa subregion. The Republic of Cameroon covers an area of 475,000 square kilometers, lying between longitudes 8° and 16° East of the Greenwich Meridian and latitudes 2° and 13° North of the equator. Seventy percent of Cameroon population is economically dependent on agriculture, essentially represented by livestock production, which is dominated by zebu and crossbreeds (European taurine × zebu and African taurine × zebu). In this context, the taurine local cattle faces extinction due to the widespread and uncontrolled crossbreeding [5]. The Goudali is the most popular of the breeds, especially in the sector of small farmers in the Adamawa highlands of Cameroon [7]. Despite its importance, few characterization studies have been conducted on the Cameroonian zebu meat [8,9]. Data were collected on live weight (LW), heart circumference (HG), height at withers (HW), trunk length (TL), age, sex, and coat color of zebu by Ebangi et al. [10], while body and meat characteristics of young bulls from Zebu Goudali of Cameroon were reported by Ojong et al. [8]. Saccà et al. compared the Goudali breed with its Italian Simmental crosses in terms of meat physical qualities and expression of some tenderness-related genes[8,9]. Genetic diversity, introgression, and relationships with *Bos taurus* were studied using genotype information on 28 markers (16 microsatellite, 7 milk protein, and 5 blood protein markers) by Ibeagha-Awemu et al. [11]. Paguem et al. characterized the whole genome of *Bos indicus indicus* breed of Cameroon focusing on their adaptive phenotypic traits and pathogen resistance [12]. 

The study of the stable isotope ratios, an innovative analytical technique, is now increasingly important in order to characterize animal products according both to their geographical origin and to the animal diet. Due to the difference in their mass number, isotopic molecules are slightly different in their physical and chemical properties. Climate, altitude, latitude, metabolism processes of the living system, and other factors influence the abundance of the isotopes (δ), providing information on the geographical origin of the products [13]. The stable isotope ratio (SIR) of bioelements—such as H, O, C, N, and S—in muscle fractions has been extensively used to assess beef origin and cattle production systems in many areas of the world [14,15,16] or to verify the preslaughter diet of beef cattle [17]. However, no information about the isotope values and their variability according to geographical distribution and animal feeding systems is available for zebu cattle reared in tropical Africa. The aim of this study was to deal with this lack of knowledge and to describe the possible use of stable isotope ratio analysis on beef fractions for zebu feeding traceability and geographical reclassification in the tropical Africa context. Sixty samples of zebu meat from the main breeds reared in Cameroon and from the areas of the greatest breeding [18] were therefore selected and analyzed for the stable isotopic compositions of C, N, S, H, and O of defatted dry matter (DFDM) and fat (FAT). The contents of saturated fatty acid (SFA), polyunsaturated fatty acid (PUFA) or monounsaturated fatty acid (MUFA) were also determined. The specimens were also classified using a three-level qualitative scale (white, cream, or yellow) of the carcass cover fat color to evaluate a possible correlation between the stable isotopic composition and the fatty acid (FA) content, which is in turn related to the nutritional status of the animal.

## 2. Results and Discussion

### 2.1. Variability of the Isotopic Signatures from Multiple Fractions and Their Use as Geographic Tracers

The carbon stable isotope ratios of Cameroonian beef defatted dry matter (δ^13^C_DFDM_ = −11.8‰ ± 1.22‰; Table 1) fall in the highest part of the range of values reported in the literature. Similar values were recorded by Schmidt et al. [19] in beef samples from USA (−12.3‰ ± 0.1‰) and Brazil (−10.0‰ ± 0.6‰), by Nakashita et al. [20] and Horacek et al. [15] for beef produced in the USA, and by Yanagi et al. and Guo et al. [16,21] in cattle tissues from some provinces of China and farms in Japan. In all cases, the authors suggested that these high δ^13^C values may have resulted from the high content of C4 plants in cattle diets. Indeed, while C3 plants range from −30 to −23‰, C4 plants have higher δ^13^C values in their tissue (between −14 and −12‰) [22,23]. The variation is mainly due to the biochemical fractionation due to plant use of CO_2_ (C3, C4, and CAM plants that have different photosynthesis pathways) that affects the carbon ratio (^13^C/^12^C) [24]. The carbon stable isotope composition of plant tissues is determined both by the isotopic composition of the CO_2_ source and by the discriminative use of the heavier isotope ^13^C during photosynthetic CO_2_ fixation. 

The zebu is a grazer, very much like a cow, and eats mainly grass, leaves, and flowers. For this reason, the low negative δ^13^C values probably reflect the almost exclusive use of tropical C4 pasture grasses [25]. Similar behavior was reported by Erasmus et al. in South African lamb fed exclusively on grass (mean −15.8‰) while the presence in the diet of shrubs and bushes, mainly composed by CAM photosynthetic plants, leads to more negative values (between −24.3 and −19.6‰) [26]. 

As already described for other types of meat [27,28], the δ^13^C values of protein and fat fractions were correlated (r_DFDM/FAT_ = 0.81; *p* < 0.01; data not reported), even if the δ^13^C mean value of the protein (−11.8‰) was significantly more positive than the same stable isotope ratio measured in the fat (−17.8‰; *p* < 0.01). This is due to the depletion in ^13^C that occurs during the oxidation of pyruvate to acetyl-CoA in the biosynthesis of lipids and to the possible enrichment in ^13^C occurring during amino acid cycling. In the fat fraction, both examined sources of variability—beef origin and subcutaneous fat color—reached the threshold of significance in their effect on δ^13^C values. 

The δ^2^H_DFDM_ values of Cameroonian zebu (−62.8‰ ± 4.99‰; Table 1) are higher than those observed by Horacek and Min in beef produced in Korea, USA, Mexico, Australia, or New Zealand [15]. These enriched hydrogen isotope ratios could be the result of both the local climate and the free-range pasture feeding. Indeed, as the 20–30% of hydrogen body protein derives from drinking water [29], the hydrogen isotopic composition of beef protein is likely to have memorized the deuterium signature of the water that the zebu consumed, which is in turn affected by the regional fractionation deriving from the global hydrological cycle.

Furthermore, the hydrogen in the carbohydrates and proteins of the feed also contributes to the total metabolic pool of hydrogen in the animals’ organism [30]. So, if cattle are fed on fresh feed, such as herbage at pasture, a significant fraction of the daily water demand is ingested with the plant material. As plant water is enriched in ^2^H compared to groundwater due to evapotranspiration processes, the δ^2^H ratio of beef will be also enriched.

As already observed in other meat types, the fat fraction was highly ^2^H depleted in comparison to the defatted muscle (mean values: −62.8‰ vs. −179.5‰, respectively, for δ^2^H_DFDM_ and δ^2^H_FAT_; *p* < 0.01), the two fractions being nevertheless correlated to each other (*r* = 0.36; *p* < 0.01; data not reported). Moreover, the regional average values of both muscle fractions were correlated to the δ^2^H of mean annual precipitation (δ^2^H_precipitation_) of some main cities of the Cameroonian regions (Ngaundéré in Adamawa, Bamenda in the North-West, and Bertoua in the West). These values were estimated by considering the average altitude, latitude, and longitude, through the prediction model available at http://wateriso.eas.purdue.edu/waterisotopes/ (average δ^2^H_precipitation_ in Ngaundéré, −29‰, Bamenda, −38‰ and Bertoua, −24‰) [31].

In the same way as deuterium, the δ^18^O_DFDM_ average value of zebu beef (17.3‰ ± 0.78‰; Table 1) is higher than that reported for beef from other areas of the world [20]. This is probably due to the geographic and climatic gradient caused by systematic global variations in the isotope composition of precipitation water, transferred to some extent to the isotopic values of beef [30]. The oxygen isotope ratios of the muscle fractions were statistically different (mean values: 17.3‰ vs. 23.6‰, respectively, for δ^18^O_DFDM_ and δ^18^O_FAT_; *p* < 0.01), probably because of the isotopic effects of their predominant oxygen functional groups (i.e., the carbonamide group for oxygen in proteins and the ester group for oxygen in triglycerides are respectively enriched in ^18^O by ~22‰ and ~28‰ as compared to cell water) [32]. The oxygen isotope ratios of the different muscle fractions were correlated to each other (*r* = 0.49%; *p <* 0.01) and to the mean isotopic composition of meteoric water (δ^18^O_precipitation_), estimated on the basis of the geographical data, using the previously cited prediction model (average δ^18^O_precipitation_ in Ngaundéré, −4.9‰; Bamenda, −6.2‰; and Bertoua, −4.4‰) [31].

The regional δ^15^N_DFDM_ means ranged between 4.5‰ (Adamawa) and 5.4‰ (Northwest, *p* < 0.05; Table 1). Geographical patterns in δ^15^N values have already been found for meat [33]. They are probably caused by the different ^15^N content of local grass on which the zebu graze, which in turn could be a consequence of climate and soil conditions in the different geographical areas. Following the positive correlation between δ^15^N_soil_ with the mean annual temperature (MAT) and the negative one with the mean annual precipitation (MAP), a regression model was proposed by Amundson et al. to use climate data to represent spatial trends in soil δ^15^N values and to follow the high correlation in plants growing in that soil. The global trends resulted in strong latitudinal banding, with high northern latitude ecosystems having the most depleted soil δ^15^N values and arid and tropical zones having the most positive soil δ^15^N values (for Cameroon between 6.2 and 7.6‰) [34]. 

The variability of the ^34^S/^32^S ratio was also influenced by the regional origin of beef (8.59‰ vs. 7.45‰, respectively, in the northwestern and eastern regions, *p* < 0.05; Table 1). The δ^34^S values generally provide product signatures on a small geographic scale [33] because this element is controlled by the local bedrock (igneous or sedimentary, acidic, or basic) and by the atmospheric deposition and it is influenced by microbial processes in the soil [35]. Njabo et al. reported similar high values in Cameroon lowland forest mosquitoes, which reflect the local diet of their hosts [36].

After having separately examined the individual variability of the isotopic signatures from multiple fractions, a multivariate approach was considered.

Stepwise discriminant analysis was performed to trace beef origin on the basis of the stable isotopic signatures of the product and to verify which isotope ratios contribute toward the suitable classification of the different meat types. The results are given in Table 2, together with the number and percentage of correctly classified observations. Four stable isotope ratios of specific fractions of matter were selected, due to their significant contribution in the discrimination of beef origin, sorted by entering them in the following order: δ^15^N_DFDM_; δ^34^S_DFDM_; δ^18^O_FAT_; δ^2^H_DFDM_. The selected model allowed the corrected empirical allocation of 81.7% of beef samples and the corrected cross-validation of 75% of individual samples. These findings are in line with those previously obtained for other meat types [17,33], even if slightly lower. This result, less clear than the previous ones, that were characterized by higher cross-validation percentages, is likely linked to the fact that some of the zebu sent by train from Ngaundéré (and thus recorded as of Adamawa origin) to Yaoundé for slaughtering could have traveled on foot from their actual original area in the East.

### 2.2. Correlation between Stable Isotope Ratios and Nutritional Status of Beef

At slaughter, in African environmental conditions, it is easy to identify the subcutaneous fat color of the carcasses. It is widely known that the fat color of beef can influence the consumers on appreciating and purchasing the product. As reviewed by Dunne et al. [37], changes in fat yellowness were often attributed to the animal diet and to the proportion of forage to concentrate, in particular because of the β-carotene content in the forage. Feeding cattle on grass can modify their beef unsaturated fatty acids [38].

In our conditions, all the zebu were exclusively fed on natural pasture (grass). The color of subcutaneous fat recorded during slaughtering was significantly related to fat content and composition (Table 3). Independently of the origin, the intramuscular fat content was low [39], but comparable with that reported in Fulani bulls raised on natural pasture [40], possibly because of the lack of genetic selection of local breeds [41]. The level of beef lipids was particularly low in white carcasses, while the carcasses with yellow subcutaneous fat showed the fattest meat. Last carcasses were also the numerically more mature and the heaviest ones (*p* < 0.05) between classes (data not tabulated). Indeed, the yellow-colored carcasses weighed 165.2 kg, while the white- and cream-colored averaged 157.2 kg. Such a condition was associated with a slight tendency for an older age of the yellow group (3 to 5 months) in comparison with cream and white ones. 

Differences in the age and in the carcass weight together with a different herbage intake by the animals could have influenced the fat content of meat and carcasses, leading thus to the various colors of subcutaneous fat in the three carcass classes. Moreover, beef from carcasses with yellow fat had a fatty acids profile characterized by the highest content of total SFA and MUFA (Table 3) and the lowest percentage of total n-3 and n-6 PUFA.

**Figure 2:**
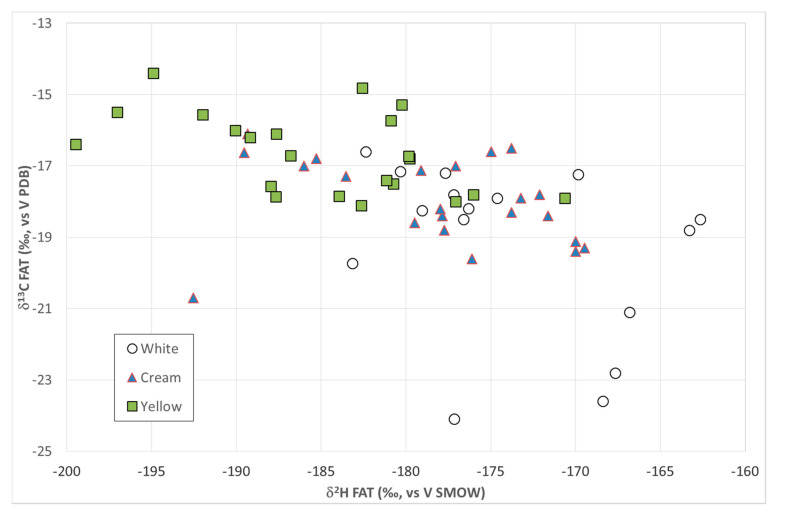
Distribution of the beef samples from zebu carcasses with different subcutaneous fat color in relationship with the stable hydrogen and carbon isotope ratios in the *longissimus thoracis* fat fraction.

The isotopic composition of the fat fraction of the muscle and, in particular, the δ^2^H[‰]_FAT_ and the δ^13^C[‰]_FAT_ values, were significantly related to the subcutaneous fat color (Table 3). As shown in Figure 2, zebu with white subcutaneous fat (“white type”) showed a clear tendency to be more enriched in ^2^H isotopes and more depleted in ^13^C isotopes than the “yellow type,” while the “cream type” represented an intermediate condition. These trends were correlated with fat composition. Indeed, ^2^H enrichment and ^13^C depletion were significantly correlated with a high PUFA content (Table 3), while ^2^H depletion and ^13^C enrichment were correlated with a high SFA content. These differences among groups could be due to the different fat content of carcasses. A lower phospholipid/neutral lipid ratio could be hypothesized for the yellow fat group, which had also the highest fat content in meat. Indeed, this group had the highest content of total SFA, mainly present in the neutral fraction, and lower content of long-chain PUFA, mainly present in the phospholipid fraction. Phospholipids, that are polar lipids, are mainly located in cell membranes, whereas neutral lipids, consisting mainly of triacylglycerol, the major constituent of reserve fat, and free fatty acids, are mainly stored in adipocytes [42]. In other words, as a consequence of better nutritional status and thus of a higher rate of subcutaneous fat deposition, the “yellow type” animals are expected to have a lower phospholipid (PL) content and a higher proportion of neutral lipids (NLs) in their subcutaneous and marble fat compared to the “white type” ones. This results in a less negative δ^13^C_FAT_ value, in agreement with other findings showing the PL fraction displaying a more negative diet-tissue fractionation than NL one [30]. 

## 3. Materials and Methods

### 3.1. Animals, Geographical Origin, Carcass Characteristics and Sample Collection

Sixty beef carcasses belonging to the most popular zebu breeds were selected and sampled among approximately 2000 cattle individually processed at the slaughterhouse of Yaounde. The 99.2% of the slaughtered cattle were zebu belonging to Goudali (GO), White Fulani (WF), and Red Mbororo (RM) breeds, and the 60% of this percentage were bulls. The majority (75%) of the cattle that were processed at the facility was raised under the transhumant pastoral system. A number of 60 zebu bulls belonging to the GO (n = 19), WF (n = 20) and RM (n = 21) breeds were selected in order to represent the animals slaughtered in terms of production system, cattle category breed, age, and carcass weight within breeds. The age at slaughter was similar for the breeds (median = 4; min. = 3; max. = 5 years). The average carcass weight of the bulls was 174.6 kg (standard error, SE = 6.58 kg) for the GO breed, 153.7 kg (SE = 4.59 kg) for WF, and 153.2 kg (SE = 5.34 kg) for RM. The experimental cattle came from three Cameroonian regions located in two ecological zones: Guinean high savannah of Adamawa and East Region and Western high plateaus of Northwest region. As recorded at slaughtering time, these bulls were raised on natural pasture [25,41], i.e. on an herbage diet based on exclusively C4 plants species such as *Hyparrhenia, Panicum,* and *Setaria* spp. [43]. 

**Figure 3:**
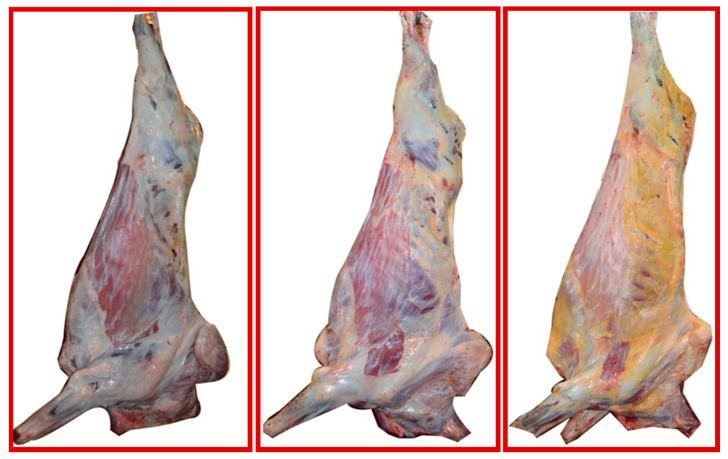
The three-level scale used for the visual assessment of the subcutaneous fat color.

The subcutaneous fat color of the carcass was visually evaluated on the lateral face of the left side, using a three-level qualitative scale (white, cream, or yellow; Figure 3). After chilling at 4 °C for 24 h, a sample of *longissimus thoracis* muscle (LT) was taken from the left side of the carcasses by cutting a 3 cm thick chop from the section between the 12th and 13th rib, but over the LT. The samples were divided in two subsamples, which were vacuum-packed, rapidly frozen, and stored at −20 °C until the time of preparation for isotopic and FA assay.

The samples were divided according to the origin and the fat color. As for the last characteristic, carcass groups showed the following attributes:

Age of bulls: yellow = 4 years (y) and 2 months (mo.); cream= 3 y, 11 mo.; white = 3 y, 9 mo.

Carcass weight: yellow = 165.2 kg (SE = 5.36 kg); cream = 157.3 kg (SE = 6.40 kg); white = 157.1 kg (SE = 5.60 kg). 

### 3.2. Sample Preparation and Analysis

#### 3.2.1. Sample Extraction and Fatty Acid Analysis

The first subsample was subject to extraction of total lipids according to [44]. In particular, after mincing, 1.5 g of meat was taken and added to nonadecanoic acid (C19:0), then homogenized in 30 mL of a chloroform–methanol mixture (2:1 *v*/*v*) using an Ultra-Turrax T 25 basic (Ika-Werke, Staufen im Breisgau, Germany). The tissue was thus filtered by vacuum filtration using Whatman filter paper. The extract was washed with 8.5 mL of 0.88% (*w*/*v*) KCl, mixed vigorously for 1 min, and then left overnight. The organic phase was separated and the solvents were evaporated under vacuum at 40 °C. FA methyl esters were prepared using HCl methanolic [45]. The lipid sample was mixed with 2 mL of hexane and 3 mL of HCl methanol in 20 mL glass tubes with Teflon-lined caps. The mixture was heated at 70 °C for 2 h in a metal block and cooled to room temperature; then, methyl esters were extracted in 2 mL of hexane after the addition of 5 mL K_2_CO_3_ of 6% (*w*/*v*) and Na_2_SO_4_. Samples stood for 30 min and were thus centrifuged. The upper hexane layer was removed, concentrated under nitrogen, then diluted in hexane and stored until measurement.

Methyl ester analysis was performed using Carlo Erba Instruments gas chromatography (HRGC 5300 mega-series, Rodano, Milano, Italy) fitted with an automatic sampler (Model A200S, Rodano, Milano, Italy) and FID detector (Rodano, Milano, Italy). A 1 μL volume of the sample was injected into the gas chromatography in split mode (split ratio: 1:30). The conditions used were the following: Omegawax-fused silica capillary column (30 m × 0.32 mm i.d., film thickness 0.25 μm) (Supelco Inc., Bellafonte, Pennsylvania, United States), programmed temperature from 160 to 240 °C at 4 °C/min and from 200 to 240 °C at 10 °C/min and then held for 5 min. The carrier gas used was helium at 1.2 mL/min flux. FA methyl esters were identified using external standards and quantified using C19:0 as the internal standard and expressed as a percentage of the total lipids identified.

#### 3.2.2. Sample Fractionation and Stable Isotope Ratio Analysis

The second subsample was minced and freeze-dried in a lyophilizer (freeze-drier), homogenized with a suitable grinder, and freeze-dried again. The resulting dry powder was divided into crude fat (FAT), through the extraction with petroleum ether for 6 h in a Soxhlet apparatus, and defatted dry matter (DFDM), essentially protein. Afterward, the DFDM and FAT fractions were stored in an appropriate container until measurement. Measurement of the ^13^C/^12^C, ^15^N/^14^N, ^2^H/^1^H, and ^18^O/^16^O ratios of DFDM and FAT fractions was carried out as described by Perini et al. [27,33]. 

The ^13^C/^12^C ^15^N/^14^N and ^32^S/^34^S ratios were measured in a single run (and weighted around 0.5 mg) using an isotope ratio mass spectrometer (IRMS) (Isoprime Ltd., Stockport, UK) following total combustion in an elemental analyzer (VARIO CUBE, Elementar Analysensysteme GmbH, Langenselbold, Germany). The ^2^H/^1^H and ^18^O/^16^O ratios were measured in a single run (around 0.5 mg) using an IRMS (Finnigan DELTA XP, Thermo Scientific, Waltham, MA, USA) coupled with a pyrolyzer (Finnigan TC/EA, high-temperature conversion elemental analyzer, Thermo Scientific).

According to the IUPAC protocol, the values are denoted in delta in relation to the international Vienna-Pee Dee Belemnite (V-PDB) for δ^13^C, Air for δ^15^N, Vienna-Canyon Diablo Troilite (V-CDT) for δ^34^S, and Vienna-Standard Mean Ocean Water (V-SMOW) for δ^18^O and δ^2^H, according to the following general equation:

(1) $$\delta i\;E=\frac{\left(i\;\mathrm{RSA}\;-\;i\;\mathrm{RREF}\right)}{i\;\mathrm{RREF}}$$

where i is the mass number of the heavier isotope of element E (for example, ^13^C), RSA is the respective isotope ratio of a sample (such as for C: number of ^13^C atoms/number of ^12^C atoms or as approximation ^13^C/^12^C), and RREF is the respective isotope ratio of internationally recognized reference material.

For δ^13^C and δ^15^N, the samples were analyzed using a single working standard for normalization, calibrated against NBS-22 fuel oil (IAEA-International Atomic Energy Agency, Vienna, Austria), IAEA-CH-6 sucrose for δ^13^C, and USGS 40 (U.S. Geological Survey, Reston, VA, USA) for both δ^13^C and δ^15^N and potassium nitrate IAEA-NO_3_ for δ^15^N. We did not use a calibration curve for δ^13^C as suggested by IUPAC [46] because as we used a single standard with a value similar to that of the samples, the data determined using a single-anchoring point or two-three anchoring points were not significantly different [47]. The δ^34^S values were calculated against barium sulfates IAEA-SO-5 (δ^34^S = +0.5‰) and NBS 127 (δ^34^S = +20.3‰) and a calibrated protein working standard through the creation of a linear equation. 

The δ^2^H and δ^18^O values of the defatted protein were calculated against Caribou Hoof Standard (CBS) (δ^2^H = −197 ± 2 ‰ and δ^18^O = +3.8 ± 0.3‰) and Kudu Horn Standard (KHS) (δ^2^H = −54 ± 1‰ and δ^18^O = +20.3 ± 0.3‰) through the creation of a linear equation and adopting the “comparative equilibration procedure” [48]. We used these two keratinous standards because of the absence of any international organic reference material with a matrix similar to ours. The δ^2^H and δ^18^O of fat were calculated against two working in-house standards (commercial olive oils) calibrated against NBS-22 fuel oil (−120‰) and magnesium stearate for the FIRMS FT method (δ^2^H value: −228‰) and benzoic acid-601 (+23.1‰) and −602 (+71.3‰) (International Atomic Energy Agency (IAEA), Vienna, Austria). One control sample was routinely included in each analytical run to check system performance and we obtained very repeatable results over the 2-month running period. Measurement uncertainty, expressed as one standard deviation when measuring a sample 10 times, was ≤2‰ for δ^2^H, 0.3‰ for δ^34^S and for δ^18^O, and 0.2‰ for δ^13^C and δ^15^N.

#### 3.2.3. Statistical Analysis

Statistical analysis of data was performed using the SPSS Statistics version 17 for Windows (SPSS Inc., Chicago, IL, USA). The data for each stable isotope ratio were summarized as mean and standard deviation values. The effect of beef origin and fat color (linked to the type of diet) on each stable isotope ratio and fatty acid profile was investigated using ANOVA. The associate variance between isotope ratios and fatty acid profile was evaluated using the Pearson correlation coefficient, r.

Canonical discriminant analysis was carried out to evaluate whether multivariate separation for classifying beef origin could be based on the stable isotopic signatures of bioelements and to verify which isotope ratios contribute toward enabling this classification. The most discriminant ratios were selected by a stepwise procedure and the significance of each discriminant function was evaluated on the basis of Wilks’ Lambda statistics. The success of discrimination was measured by the proportion of observations incorrectly allocated to groups, using 10-fold cross-validation.

## 4. Conclusions

Zebu beef from Cameroon showed a specific isotope profile, characterized by higher δ^13^C, δ^2^H, and δ^18^O values than those reported in other areas of the world, as a consequence of the almost exclusive use of tropical C4 pasture grasses for cattle feed and the geographic and climatic gradient in the isotopic composition of precipitation water.

The isotopic composition of the fat fraction of muscle was significantly linked to the subcutaneous fat color. Zebu with white subcutaneous fat (“white type”) showed a clear tendency to be more enriched in ^2^H isotopes and more depleted in ^13^C isotopes than the “yellow type,” while the “cream type” represented an intermediate condition. These trends correlated with fat composition: ^2^H enrichment and ^13^C depletion were significantly correlated with a high PUFA content, while ^2^H depletion and ^13^C enrichment were correlated with a high SFA content. It was argued that, as a consequence of better nutritional status, the “yellow type” animals had a lower phospholipid (PL) content and a higher proportion of neutral lipids (NL) in their fat compared to the “white type” ones. This results therefore in less negative δ^13^C_FAT_ values, in agreement with the findings showing that the PL fraction displays a more negative diet-tissue fractionation than the NL one.

Within the country, multielement stable isotope ratio analysis gave promising results for tracing the regional origin of beef and some aspects of the cattle breeding system, such as the animal’s nutritional status.

## Figures and Tables

**Table 1:** Stable isotope ratios (observed mean and standard deviation).

Fat Color	Region	δ^13^C_[‰]DFDM_^i^		δ^13^C_[‰]FAT_^i^		δ^2^H_[‰]DFDM_		δ^2^H_[‰]FAT_		δ^18^O_[‰]DFDM_		δ^18^O_[‰]FAT_		δ^15^N_[‰]DFDM_		δ^34^S_[‰]DFDM_	
		Mean	SD	Mean	SD	Mean	SD	Mean	SD	Mean	SD	Mean	SD	Mean	SD	Mean	SD
**White**	Adamawa	−12.2	1.44	−18.7	1.87	−60.5	4.99	−174.1	6.30	17.4	0.90	23.7	1.26	4.70	0.58	8.09	0.84
	Northwest	−13.1	2.55	−20.9	2.53	−60.9	7.36	−172.4	7.70	17.3	0.97	22.2	0.96	5.80	0.73	8.98	1.27
	East	−11.3	0.70	−17.0	0.36	−66.8	5.67	−176.6	6.33	17.3	0.44	24.6	0.14	4.70	0.89	7.39	0.50
	Total	−12.4	1.89	−19.2 ^A^	2.39	−61.8	6.19	−173.9 ^B^	6.58	17.4	0.82	23.3	1.35	5.11	0.85	8.30	1.11
**Cream**	Adamawa	−11.5	0.78	−18.1	1.32	−63.2	4.23	−177.9	7.03	17.4	0.52	23.8	1.31	4.53	0.80	7.86	0.88
	Northwest	−11.7	1.10	−17.7	0.99	−61.1	0.81	−178.8	10.16	17.3	0.85	22.2	0.30	5.25	0.06	8.32	0.49
	East	−12.3	0.90	−17.6	0.98	−65.4	0.94	−178.8	6.57	16.9	0.75	24.2	1.49	4.81	0.48	7.16	0.38
	Total	−11.7	0.85	−18.0 ^AB^	1.21	−63.5	3.69	−178.2 ^AB^	6.80	17.3	0.61	23.7	1.36	4.66	0.72	7.74	0.82
**Yellow**	Adamawa	−11.3	0.67	−16.6	1.11	−62.8	5.71	−185.2	7.73	17.4	0.92	23.5	1.14	4.31	0.62	7.73	0.57
	Northwest	−12.0	0.30	−17.7	0.19	−64.2	3.17	−186.0	2.84	16.3	0.49	21.7	0.29	5.16	0.33	8.48	0.21
	East	−11.6	1.15	−15.8	1.34	−60.5	2.35	−181.2	1.94	17.9	0.35	26.9	0.18	4.33	1.12	7.79	0.87
	Total	−11.4	0.70	−16.6 ^B^	1.12	−62.7	5.28	−184.9 ^A^	7.10	17.3	0.92	23.6	1.56	4.39	0.66	7.80	0.59
Total	Adamawa	−11.5	0.92	−17.6 ^AB^	1.58	−62.5	5.04	−180.5	8.37	17.4	0.77	23.6 ^B^	1.20	4.46 ^b^	0.69	7.84 ^b^	0.74
	Northwest	−12.6	2.06	−19.6 ^B^	2.52	−61.6	5.76	−176.4	8.80	17.1	0.90	22.1 ^C^	0.76	5.56 ^a^	0.64	8.75 ^a^	1.01
	East	−11.9	0.93	−17.1 ^A^	1.09	−64.8	3.71	−178.6	5.60	17.2	0.68	24.8 ^A^	1.48	4.68 ^b^	0.68	7.35 ^b^	0.52
	Total	−11.8	1.22	−17.8	1.86	−62.8	4.99	−179.5	8.08	17.3	0.78	23.6	1.42	4.68	0.78	7.91	0.86

^i^: DFDM: *longissimus thoracis* defatted dry matter; FAT: *longissimus thoracis* crude fat. ^a,b,c^: means of the levels of a factor (on the column) with different superscript letters differ at *p* ≤ 0.05. ^A,B,C^: means of the levels of a factor (on the column) with different superscript letters differ at *p* ≤ 0.01.

**Table 2:** Results (%) of the best reclassification of beef samples from zebu of different origin on the basis of the linear discriminant functions calculated from the stable isotope data.

	Region	Predicted Beef Origin		
		Adamawa	Northwest	East
Original ^i^	Adamawa	75.0	12.5	12.5
	Northwest	0.0	100.0	0.0
	East	0.0	10.0	90.0
Cross-validated ^ii^	Adamawa	65.0	17.5	17.5
	Northwest	0.0	100.0	0.0
	East	0.0	10.0	90.0

^i^: 81.7% of original grouped cases correctly classified. ^ii^: 75.0% of cross-validated grouped cases correctly classified.

**Table 3:** Relationship of *longissimus thoracis* fat content (Total lipids, TL, % dry matter) and fatty acid profile (%TL) with subcutaneous fat color and isotope composition of *longissimus thoracis* fat fraction.

	Fatty Acid Profile				Correlation	
Subcutaneous Fat Color:	White	Cream	Yellow	SE	δ^2^H[‰]_FAT_	δ^13^C[‰]_FAT_
no. of samples	16	22	22			
Total lipids	3.3 ^b^	4.1 ^b^	7.1 ^a^	2.61	−0.478 **	0.413 **
SFA	47.1 ^b^	49.5 ^ab^	52.2 ^a^	5.12	−0.514 **	0.335 **
MUFA	33.1 ^b^	35.3 ^b^	38.3 ^a^	4.21	−0.315 *	0.273 *
PUFA-n3	6.4 ^a^	5.0 ^b^	3.2 ^c^	1.57	0.655 **	−0.466 **
PUFA-n6	13.4 ^a^	10.2 ^b^	6.2 ^c^	3.50	0.675 **	−0.506 **
PUFA	19.8 ^a^	15.2 ^b^	9.4 ^c^	4.95	0.665 **	−0.483 **

^a,b,c^: means on the row with different superscript letters differ at *p* ≤ 0.05. *, **: correlation significance *p* ≤ 0.05 and *p* ≤ 0.01, respectively.

## Data Availability

The data presented in this study are available on the request from the corresponding author.

## Sample Availability

Samples of the compounds are not available from the authors.

## Abbreviations

*Longissimus thoracis* muscle (LT); defatted dry matter (DFDM); fatty acid (FA); fat (FAT); saturated fatty acid (SFA), polyunsaturated fatty acid (PUFA); monounsaturated fatty acid (MUFA); isotope ratio mass spectrometry (IRMS); mean annual temperature (MAT); mean annual precipitation (MAP).
